# Two new species of *Tremella arisaematis* (*Tremellaceae*) and *Pseudotremella guizhouensis* (*Bulleraceae*) from China

**DOI:** 10.3897/mycokeys.141.205833

**Published:** 2026-09-30

**Authors:** Long Tian, Chun-Yue Chai, Qiu-Hong Niu, Feng-Li Hui

**Affiliations:** 1 School of Life Science, Nanyang Normal University, Nanyang 473061, China School of Life Science, Nanyang Normal University Nanyang China; 2 State Key Laboratory of Motor Vehicle Biofuel Technology, Nanyang 473000, China State Key Laboratory of Motor Vehicle Biofuel Technology Nanyang China

**Keywords:** Basidiomycetous yeast, plant leaf, rotting wood, *
Tremellales
*

## Abstract

In the present study, three basidiomycetous yeast strains were isolated from plant materials collected in Guizhou and Henan provinces, China. Phenotypic characteristics, together with phylogenetic analyses based on the D1/D2 domain of the large subunit (LSU) rRNA gene and the internal transcribed spacer (ITS) region, were used to characterize and identify these isolates. As a result, two novel species, namely *Tremella arisaematis* sp. nov. and *Pseudotremella guizhouensis* sp. nov., are described herein. In the phylogeny inferred from the concatenated D1/D2 and ITS sequences, *T. arisaematis* sp. nov. was closely related to *T. saccharicola*, whereas *P. guizhouensis* sp. nov. was resolved as a sister species to *P. allantoinivorans*. Illustrated descriptions of the two novel species and comparisons with closely related taxa are provided. These results indicate that the species’ diversity of *Tremella* and *Pseudotremella* remains underexplored, with additional undescribed taxa likely awaiting discovery.

## Introduction

*Tremella*, validly described by Persoon in 1794, is the largest, albeit polyphyletic, genus in the order *Tremellales* (Bandoni and Boekhout 2011; Weiss et al. 2014; Zhao et al. 2019). As traditionally circumscribed, *Tremella* sensu lato comprises approximately 90 species, more than half of which are recognized as lichenicolous taxa and exhibit a high degree of host specialization (Bandoni and Boekhout 2011; Millanes et al. 2011, 2012, 2014, 2015, 2016; Zamora et al. 2011, 2016; Diederich et al. 2014; Kout et al. 2015; Lindgren et al. 2015; Westberg et al. 2015; Spirin et al. 2017; Zhao et al. 2019; Fan et al. 2021). Phylogenetic analyses have shown that *Tremella* s.l. can currently be divided into four major clades: *Tremella* sensu stricto and three additional clades comprising lichenicolous species, provisionally designated as *Tremella* clades I, II, and III. (Millanes et al. 2011; Liu et al. 2015a, 2015b). However, the taxonomy of these lineages remains unresolved pending more robust phylogenetic evidence and the discovery of additional diagnostic morphological characters. *Tremella* s.s. is currently restricted to the *Fuciformis* and *Mesenterica* subclades, which together include more than 30 species, over half of which are known to possess a yeast state (Khunnamwong et al. 2019; Fan et al. 2021; Navarro et al. 2022). The life cycle of *Tremella* s.s. species comprises two distinct stages: a teleomorphic, diploid stage, in which basidiocarps are formed on suitable substrates under appropriate conditions, and an anamorphic, haploid stage, in which basidiospores germinate by budding and develop as haploid yeast cells (Bandoni and Boekhout 2011; Weiss et al. 2014; Khunnamwong et al. 2019).

*Pseudotremella* was established by Liu et al. (2015b) to accommodate four species, namely *P. allantoinivorans*, *P. lacticolor*, *P. moriformis*, and *P. nivalis*, which had previously been recognized as members of the moriformis clade within the *Tremellales* (Chen 1998; Satoh et al. 2013; Liu et al. 2015a, 2015b). Subsequently, *P. hippophaes*, *P. lichenophila*, *P. navarinensis*, and *P. rhododendri* were described as four additional species of the genus *Pseudotremella* (Jiang et al. 2024; Pérez et al. 2024; Feng et al. 2025). To date, the genus *Pseudotremella* comprises eight species. Among them, only *P. moriformis* and *P. nivalis* have been reported to possess known sexual morphs, which occur on natural substrates and form tremelloid basidiocarps and basidia (Bandoni and Boekhout 2011; Liu et al. 2015b). The remaining representatives of the genus are known only from asexual morphs resembling yeast states and reproduce by budding; some species may also produce pseudohyphae and/or true hyphae. Physiologically, species of *Pseudotremella* do not ferment sugars or assimilate methanol, and they usually show positive reactions in diazonium blue B (DBB) and urease tests (Middelhoven 2005; Liu et al. 2015b; Jiang et al. 2024; Pérez et al. 2024; Feng et al. 2025).

During a survey of yeast diversity in China, three yeast isolates were obtained from plant leaves and rotting wood collected in Guizhou and Henan provinces. Molecular phylogenetic analyses based on the D1/D2 domain of the large subunit (LSU) rRNA gene and the internal transcribed spacer (ITS) region, together with phenotypic characterization, revealed that these isolates represent two novel species belonging to the genera *Tremella* and *Pseudotremella*. The novel taxa are described and illustrated herein.

## Materials and methods

### Sample collection and yeast isolation

The yeast strains analyzed in this study were isolated from plant leaves collected in the Baotianman Nature Reserve, Neixiang County, Henan Province, China (33°30'N, 111°55'E), and from rotting wood samples collected in Pingtang Wetland Park, Pingtang County, Guizhou Province, China (25°29'N, 106°40'E). Each sample was placed in a sterile plastic bag, kept on ice, and transported to the laboratory within 24 hours for subsequent processing. Isolates from plant leaves were obtained using the ballistoconidia-fall technique (Nakase and Takashima 1993), whereas those from rotting wood samples were recovered following previously described protocols (Gao et al. 2021). All isolates were purified and preserved in 20% glycerol at −80 °C for long-term storage in our laboratory.

### Phenotypic characterization

Morphological characteristics and physiological and biochemical properties were examined using the standard methods described by Kurtzman et al. (2011). Colony and cell morphologies were observed after 3 and 7 days of growth, respectively, on YM agar (YM; 0.3% yeast extract, 0.3% malt extract, 0.5% peptone, 1% glucose, and 2% agar) at 20 °C. The formation of hyphae and pseudohyphae was examined on Dalmau plates prepared with corn meal agar (CMA; 2% cornmeal infusion and 2% agar) after incubation at 20 °C for 2 weeks. Ballistoconidium formation was tested using the inverted-plate method (do Carmo-Sousa and Phaff 1962) on CMA at 15 and 20 °C for 2 weeks, and discharged spores were examined microscopically after 3–14 days. To induce sexual reproduction, individual strains and strain pairs were incubated at 17 °C on three different media, namely CMA, potato dextrose agar (PDA; 20% potato infusion, 2% glucose, and 2% agar), and V8 agar (10% V8 juice and 2% agar), for up to 2 months, with microscopic observations conducted biweekly (Khunnamwong et al. 2019; Li et al. 2020). Carbon and nitrogen source assimilation tests were performed in liquid media, and starved inocula were used for nitrogen assimilation assays. Glucose fermentation was evaluated using Durham fermentation tubes. Temperature tolerance was assessed by incubating the strains on YM agar at 15, 20, 25, 30, 35, and 37 °C for 7 days. The newly proposed taxa were registered in the MycoBank database (https://www.mycobank.org; accessed 26 June 2026).

### DNA extraction, PCR amplification, sequencing

Genomic DNA was extracted from yeast cells using the Ezup Column Yeast Genomic DNA Purification Kit (Sangon Biotech, Shanghai, China) according to the manufacturer’s instructions. The D1/D2 domain of the LSU rRNA gene and the ITS region were amplified using the primer pairs NL1/NL4 (Kurtzman and Robnett 1998) and ITS1/ITS4 (White et al. 1990), respectively. PCR amplifications were performed in 25 μL reaction mixtures containing 9.5 μL nuclease-free water, 12.5 μL 2× Taq PCR Master Mix with blue dye (Sangon Biotech, Shanghai, China), 1 μL genomic DNA template, and 1 μL of each primer. The PCR conditions were as follows: initial denaturation at 98 °C for 2 min; followed by 35 cycles of denaturation at 98 °C for 10 s, annealing at 55 °C for 10 s, and extension at 72 °C for 15 s; and a final extension at 72 °C for 5 min. PCR products were sequenced bidirectionally by Sangon Biotech. All newly generated sequences were assembled and edited using BioEdit v.7.1.3.0 (Hall 1999). The new sequences were deposited in GenBank, and the corresponding accession numbers are provided in Tables 1, 2.

**Table 1.** Sequences used in phylogenetic analysis of *Tremella* s.s. Entries in bold are newly generated in this study.

**Species**	**Strain/clone no**.	**Locality**	**GenBank accession no**.	
			**D1/D2**	**ITS**
*Pseudotremella nivalis*	CBS 8487^T^	Taiwan, China	AF042232	AF042414
*Pseudotremella moriformis*	CBS 7810^T^	UK	AF075493	AF444331
***Tremella arisaematis***	**NYNU 2211236^T^**	**China**	**OP954734**	**OP954740**
***Tremella arisaematis***	**NYNU 2211363**	**China**	**PZ538948**	**PZ538949**
*Tremella basidiomaticola*	CGMCC 2.5725^T^	China	MH712785	MH712821
*Tremella brasiliensis*	CBS 6966^T^	—	NG_042360	NR_119454
*Tremella cerebriformis*	ZRL20170101	Taiwan, China	MH712787	MH712823
*Tremella cheejenii*	GX20172598	China	MH712789	MH712825
*Tremella cinnabarina*	CBS 8234^T^	Australia	NG_057662	NR_155933
*Tremella coalescens*	CBS 6967^T^	USA	NG_069418	NG_069418
*Tremella dysenterica*	LE 303447	Brazil	KP986542	KP986509
*Tremella erythrina*	GX20170141	China	MH712791	MH712827
*Tremella fibulifera*	211759	Brazil	MT425190	MT445850
*Tremella flava*	CBS 8471^T^	Taiwan, China	KY109891	NR_155935
*Tremella fuciformis*	CBS 6970^T^	Brazil	AF075476	AF444316
*Tremella globispora*	CBS 6972^T^	UK	NG_057771	NR_155889
*Tremella lloydiae-candidae*	VLA M-11702	—	KP986559	KP986536
*Tremella mesenterica*	CBS 6973 ^T^	Sweden	NG_069419	NR_155937
*Tremella resupinata*	CBS 8488^T^	Taiwan, China	AF042239	AF042421
*Tremella saccharicola*	DMKU SP23^T^	Thailand	NG_228714	NR_186905
*Tremella samoensis*	LE 303465^T^	Ogasawara-shoto	KP986541	KP986508
*Tremella shuangheensis*	CGMCC 2.5615^T^	China	MK050285	NR_174730
*Tremella taiwanensis*	CBS 8479 ^T^	Taiwan, China	AF042230	AF042412
*Tremella tropica*	CBS 8483^T^	Taiwan, China	NG_058416	NR_155938
*Tremella yokohamensis*	CBS 11776^T^	Japan	NG_246374	HM222926
Fungal sp.	OTU3529	Japan	MT596025	—
Uncultured fungus	AC_2d_C09	Austria	JF449469	JF449469
*Vishniacozyma taibaiensis*	CBS 9919^T^	China	NG_058434	NR_1448102

Note. Strains marked with ^‘T’^ are ex-type.

**Table 2.** Sequences used in phylogenetic analysis of *Pseudotremella*. Entries in bold are newly generated in this study.

**Species**	**Strain no**.	**Locality**	**GenBank accession no**.	
			**D1/D2**	**ITS**
*Tremella caloplacae*	AM31	Greece	JN043573	JN053468
*Tremella candelariellae*	AM34	Luxembourg	JN043575	JN053470
*Tremella exigua*	RJB6623-15	France	AF042248	AF042430
*Pseudotremella allantoinivorans*	CBS 9604^T^	Netherlands	NG_057722	NR_137662
***Pseudotremella guizhouensis***	**NYNU 25823^T^**	**China**	**PX765644**	**PX810227**
*Pseudotremella hippophaes*	CGMCC 2.6838^T^	China	OP470189	NR_200470
*Pseudotremella lacticolor*	CBS 10915^T^	Japan	NG_060058	NR_158875
*Pseudotremella lichenophila*	CPCC 300091^T^	Antarctica	OM980472	OM980383
*Pseudotremella moriformis*	CBS 7810^T^	UK	AF075493	AF444331
*Pseudotremella navarinensis*	N129_B01^T^	Magellanes	PQ362941	OQ448493
*Pseudotremella nivalis*	CBS 8487^T^	Taiwan, China	AF042232	AF042414
*Pseudotremella rhododendri*	CGMCC 2.6854^T^	China	OP470186	NR_200469
*Pseudotremella* sp.	MUCC1838	Japan	LC715711	—
*Pseudotremella* sp.	AY1076F	Portugal	MN100994	—
*Pseudotremella* sp.	CBL1-Y3	China	PZ245517	PZ263636
‘*Tremella*’ sp.	KBP Y-6774	Russia	MZ666407	MZ666407
*Pseudotremella* sp.	KBP Y-6836	Russia	ON263265	ON263265
*Pseudotremella* sp.	KBP Y-6886	Russia	OP068305	OP068305
*Bullera alba*	CBS 500^T^	Canada	NG_042387	NR_111083
*Bullera unica*	CBS 8290^T^	New Zealand	NG_058615	NR_0732562

Note. Strains marked with ^‘T’^ are ex-type.

### Phylogenetic analyses

Sequences generated in this study, together with reference sequences retrieved from GenBank (Tables 1, 2), were used for phylogenetic analyses. Reference sequences were selected based on BLAST search results and previously published datasets (Navarro et al. 2022; Feng et al. 2025). Sequences of individual loci were aligned separately using MAFFT v.7 (Katoh and Standley 2013) with default parameters and manually adjusted where necessary in MEGA v.11 (Tamura et al. 2021). The alignments of each locus were concatenated using PhyloSuite v.1.2.3 (Zhang et al. 2020).

Phylogenetic relationships were inferred using maximum likelihood (ML) and Bayesian inference (BI) analyses based on the concatenated D1/D2 and ITS sequence dataset. The ML analysis was performed using RAxML v.8.2.3 with 1,000 rapid bootstrap replicates (Stamatakis 2014). The BI analysis was conducted using MrBayes v.3.2.7a with 5,000,000 generations (Ronquist et al. 2012). The best-fit nucleotide substitution model was estimated using Modeltest v.3.04 (Kalyaanamoorthy et al. 2017), and the GTR + I + G model was selected for both ML and BI analyses. Nodes with maximum likelihood bootstrap percentages (MLBP) above 50% and Bayesian posterior probabilities (BPP) above 0.95 were considered supported.

## Results

### Molecular phylogeny

**Figure 1.**
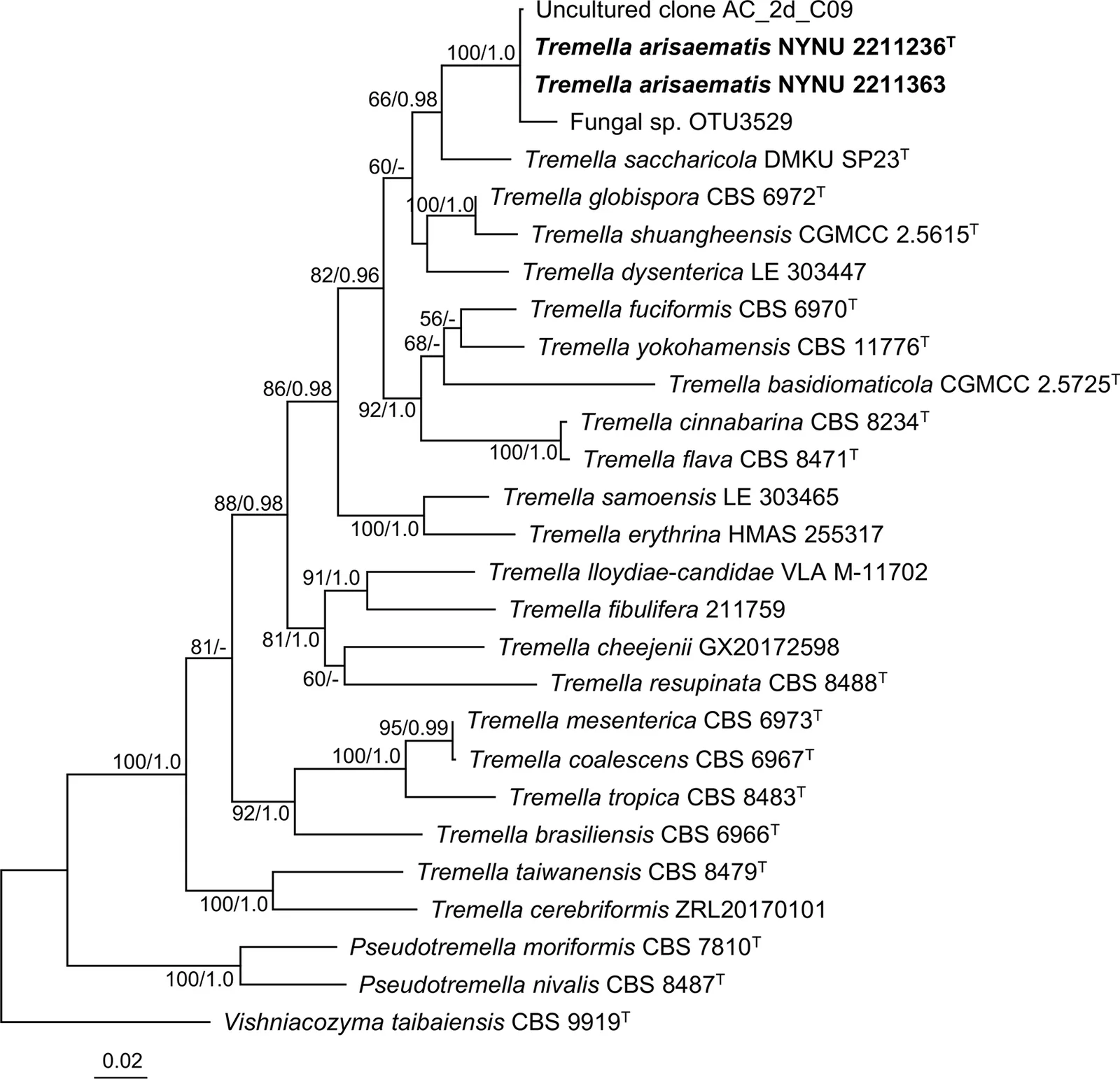
Maximum likelihood phylogenetic tree of *Tremella* s.s. based on the concatenated D1/D2 and ITS sequences. *Vishniacozyma taibaiensis*CBS 9919 was used as the outgroup. Maximum likelihood bootstrap percentages (MLBP) ≥ 50% and Bayesian posterior probability (BPP) ≥ 0.95 are shown on the branches. Type strains are denoted with the superscript ‘T’. Strains isolated in this study were marked in bold. Scale bar: 0.02 substitutions per nucleotide position.

**Figure 2.**
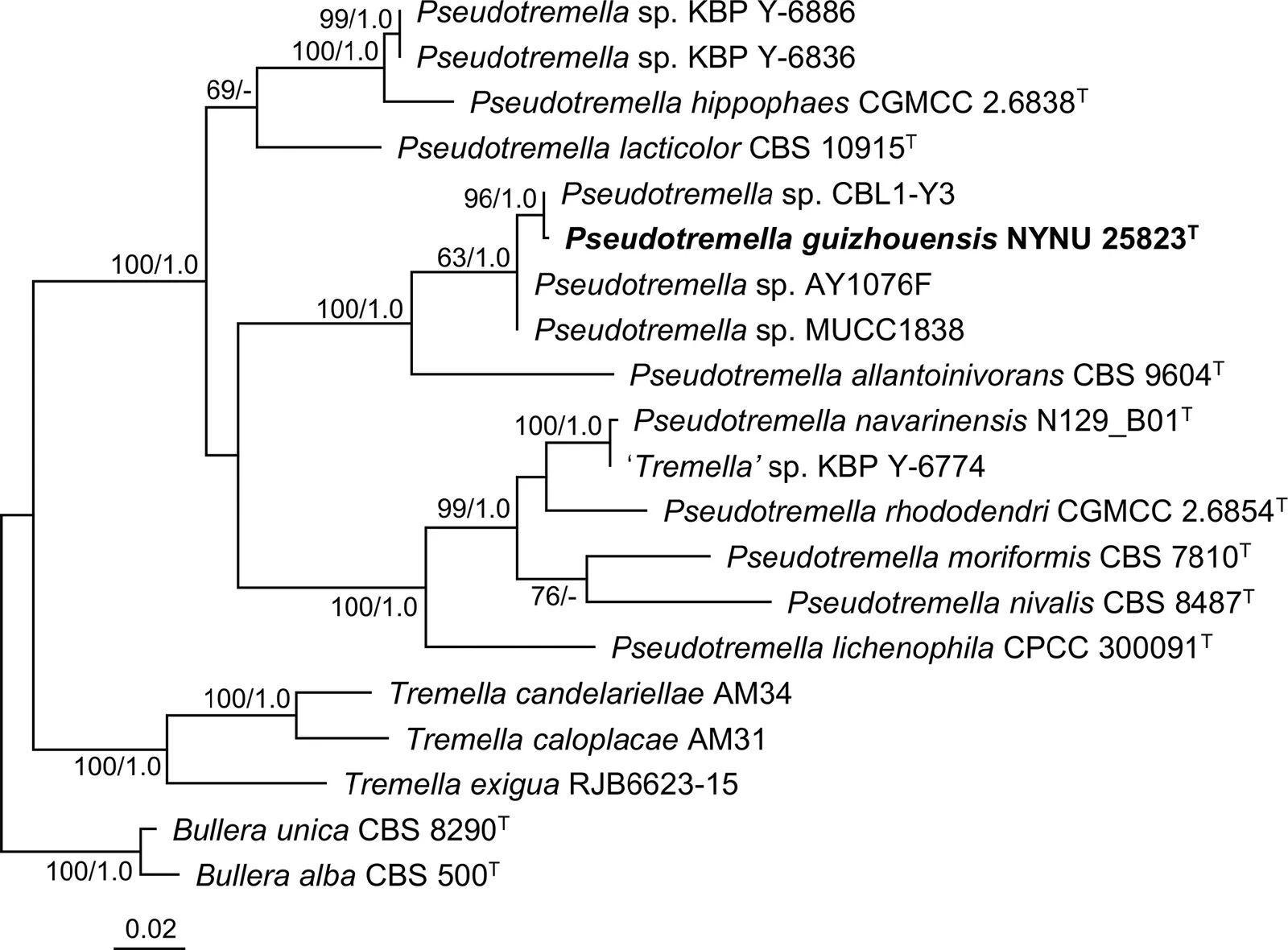
Maximum likelihood phylogenetic tree of *Pseudotremella* based on the concatenated D1/D2 and ITS sequences. The species *Bullera alba*CBS 500 and *Bullera unica*CBS 8290 were used as the outgroups. Maximum likelihood bootstrap percentages (MLBP) ≥ 50% and Bayesian posterior probability (BPP) ≥ 0.95 are shown on the branches. Type strains are denoted with the superscript ‘T’. Strains isolated in this study were marked in bold. Scale bar: 0.02 substitutions per nucleotide position.

In this study, 26 yeast strains were isolated from the collected plant samples. Based on D1/D2 and ITS sequence analyses, these yeast strains were assigned to eight known species—*Bullera alba*, *Derxomyces melastomatis*, *Filobasidium magnum*, *Hannaella phyllophila*, *Saitozyma flavus*, *Symmetrospora gracilis*, *Symmetrospora symmetrica*, *Tilletiopsis washingtonensis*—and two *Tremellales* taxa that have not yet been formally described and, therefore, likely represent two potential new species. To determine the phylogenetic positions of these two putative novel species, phylogenetic analyses were performed based on the concatenated D1/D2 and ITS sequence dataset. The resulting phylogenetic trees consistently indicated that the three strains represented two distinct novel species belonging to the genera *Tremella* and *Pseudotremella*, respectively (Figs 1, 2).

Strains NYNU 2211236 and NYNU 2211363, isolated from Henan Province, formed a well-supported clade together with strain OTU3529 from Japan and clone AC_2d_C09 from Austria within the genus *Tremella* (Fig. 1). BLASTn results showed that NYNU 2211236 and NYNU 2211363 had ITS sequences identical to those of strain OTU3529 and clone AC_2d_C09, and D1/D2 sequences highly similar to that of clone AC_2d_C09, differing by only one nucleotide, suggesting that they are conspecific. The clade represented by NYNU 2211236 was closely related to *T. saccharicola*, but differed from the type strain of *T. saccharicola* by 14 nucleotide substitutions (~2.3%) and 31 nucleotide mismatches (~6.6%) in the D1/D2 and ITS regions, respectively. These molecular differences support the recognition of the NYNU 2211236 clade as a novel species of *Tremella*, for which the name *Tremella arisaematis* sp. nov. is proposed.

Strain NYNU 25823, isolated from Guizhou Province, was placed in a distinct clade together with strains MUCC1838 from Japan, AY1076F from Portugal, and CBL1-Y3 from the Tibet Autonomous Region, within the genus *Pseudotremella* (Fig. 2). The strains within this clade differed from each other by only one nucleotide in the D1/D2 domain. Where ITS sequences were available for comparison, strains NYNU 25823 and CBL1-Y3 showed highly similar ITS sequences, with no more than two nucleotide mismatches. Compared with its closest known relative, *P. allantoinivorans* (Fig. 2), the clade represented by NYNU 25823 differed by 23 nucleotide substitutions (~3.8%) in the D1/D2 domain and 57 nucleotide mismatches (~9.8%) in the ITS region, respectively. These levels of sequence divergence support the recognition of the NYNU 25823 clade as a novel species of *Pseudotremella*, for which the name *Pseudotremella guizhouensis* sp. nov. is proposed.

### Taxonomy

#### Tremella arisaematis

Taxon classificationFungiTremellalesTremellaceae

C.Y. Chai & F.L. Hui sp. nov.

580E2291-DA50-5FFF-8829-BBF41CEBA26B

864363

Fig. 3A, B

##### Etymology.

The specific epithet arisaematis refers to *Arisaema*, the plant genus from which the type strain was isolated.

**Figure 3.**
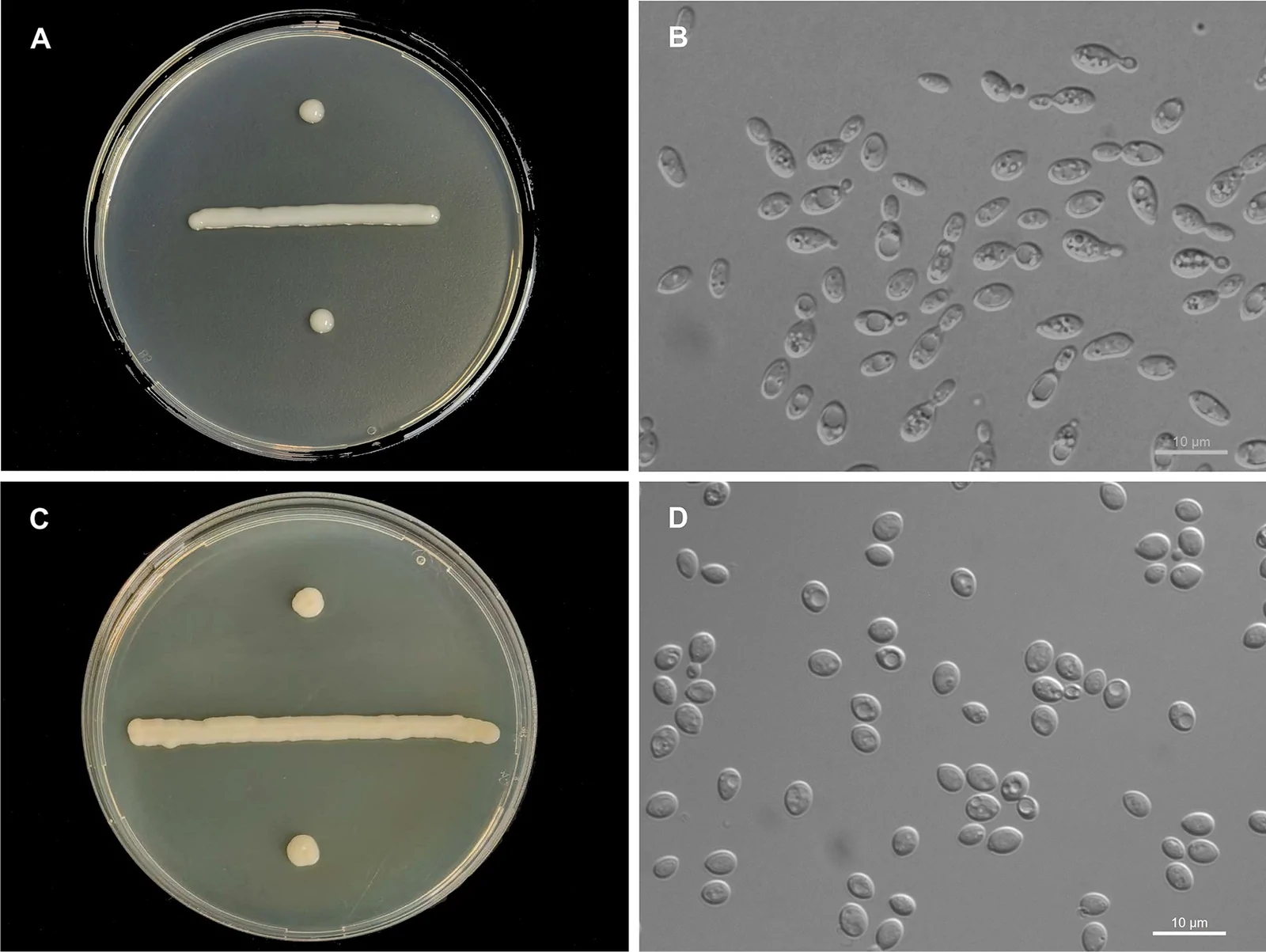
The streak culture grown on YM agar for 7 d at 20 °C and budding cells grown on YM agar for 3 d at 20 °C. **A**, **B**. *T. arisaematis* NYNU 2211236; **C**, **D**. *P. guizhouensis* NYNU 25823.

##### Typus.

China • Henan Province, Neixiang County, Baotianman Nature Reserve, on a leaf of *Arisaema heterophyllum*, September 2022, J.Z. Li, GDMCC 2.238, holotype (preserved in a metabolically inactive state). Ex-type living culture PYCC 10000 = NYNU 2211236.

##### Description.

On YM agar after 7 days at 20 °C, the streak culture is cream to light brown, mucoid, smooth and glossy with an entire margin. On YM agar after 3 days at 20 °C, cells are ellipsoidal (2.9–4.4 × 5.3–6.2 µm) and occur singly or in pairs. Budding is polar. After a month in YM broth at 20 °C, a ring and a sediment are formed. In Dalmau plate culture on CMA at 20 °C, pseudohyphae and hyphae are not formed. Sexual structures are not observed on PDA, CMA or V8 agar at 17 °C. On corn meal agar, ballistoconidia are not produced. Glucose fermentation is absent. The following carbon sources are assimilated: glucose, inulin, sucrose, raffinose, melibiose (delayed), D-galactose, lactose, trehalose, maltose, melezitose, methyl-α-D-glucoside, cellobiose, salicin (weak), L-sorbose, D-xylose, L-arabinose, D-arabinose, d-ribose, glycerol, erythritol, ribitol (weak), galactitol (weak), D-mannitol, D-glucitol, DL-lactate (delayed), succinate, citrate (delayed and weak), D-gluconate (delayed), D-glucosamine, N-acetyl-D-glucosamine, 2-keto-d-gluconate (delayed and weak), D-glucuronate (delayed), and D-glucono-1,5-lactone (weak). L-Rhamnose, methanol, ethanol, and *myo*-inositol are not assimilated. Ethylamine (weak) and L-lysine are assimilated as sole nitrogen sources. Nitrate, nitrite, and cadaverine are not assimilated. Growth is observed at 25 °C, but not at 30 °C. Growth occurs on 50% (w/w) glucose-yeast extract agar. Starch-like compounds are not produced. Urease activity and diazonium blue B reaction are positive.

##### Additional strain examined.

China • Henan Province, Neixiang County, Baotianman Nature Reserve, on a leaf of *Arisaema heterophyllum*, September 2022, J.Z. Li, NYNU 2211363.

##### GenBank accession numbers.

Holotype GDMCC 2.238 (D1/D2: OP954734, ITS: OP954740); additional strain NYNU 2211363 (D1/D2: PZ538948, ITS: PZ538949).

##### Note.

Physiologically, *T. arisaematis* can be distinguished from its close relative *T. saccharicola* by its assimilation of DL-lactate, its failure to assimilate L-rhamnose, myo-inositol, and cadaverine, and its inability to grow at 30 °C, a condition that is supported by *T. saccharicola* (Table 3).

**Table 3.** Physiological and biochemical characteristics differentiating the new species from their closely related taxa.

**Characteristic**	***T. arisaematis***	*T. saccharicola* ^*^	***P. guizhouensis***	*P. allantoinivorans* ^*^
Assimilation				
Inulin	+	s	w	–
Melibiose	d	s	–	+
L-Rhamnose	–	s	+	+
Myo-inositol	–	s	d, w	+
DL-Lactate	d	–	+	+
Citrate	d, w	+	–	+
Cadaverine	–	+	d, w	N
Growth tests				
Growth at 30 °C	–	+	–	S

Note. ^*^Data for reference species were taken from Middelhoven (2005) and Khunnamwong et al. (2019). +, positive; –, negative; d, delayed positive; s, slow; v, variable; w, weakly positive; n, not available.

#### Pseudotremella guizhouensis

Taxon classificationFungiTremellalesBulleraceae

C.Y. Chai & F.L. Hui sp. nov.

75EB7E19-2858-5833-9D19-E02500F71F79

864364

Fig. 3C, D

##### Etymology.

The specific epithet guizhouensis refers to the geographic origin of the ex-type strain, Guizhou Province.

##### Typus.

China • Guizhou Province, Pingtang County, Pingtang Wetland Park, in rotting wood, August 2025, D. Lu, GDMCC 2.577, holotype (preserved in a metabolically inactive state). Ex-type living culture JCM 38548 = NYNU 25823.

##### Description.

On YM agar after 7 days at 20 °C, streak cultures are yellowish-cream, smooth and butyrous, and the margin is entire. On YM agar after 3 days at 20 °C, cells are oval (4.0–7.6 × 4.0–8.4 µm) and occur singly or in pairs. Budding is polar. After a month in YM broth at 20 °C, a sediment and a ring are formed. In Dalmau plate culture on CMA at 20 °C, pseudohyphae and hyphae are absent. Sexual structures are not observed on PDA, CMA or V8 agar at 17 °C. On corn meal agar, ballistoconidia are not produced. Glucose fermentation is absent. The following carbon sources are assimilated: glucose, inulin (weak), sucrose (weak), raffinose, galactose (weak), lactose (weak and delayed), trehalose, D-maltose, melezitose, methyl-α-D-glucoside, cellobiose, salicin (weak), L-rhamnose, D-xylose (weak), L-arabinose, D-arabinose, D-ribose (weak), ethanol, glycerol, erythritol, ribitol (weak), galactitol, D-mannitol, D-glucitol, *myo*-inositol (weak and delayed), DL-lactate, succinate, D-gluconate, D-glucosamine (weak and delayed), N-acetyl-D-glucosamine (weak), 2-keto-D-gluconate (weak), D-glucuronate (weak), and D-glucono-1,5-lactone (weak and delayed). Melibiose, L-sorbose, methanol, and citrate are not assimilated. Nitrate (weak), nitrite, L-lysine and cadaverine (weak and delayed) are assimilated as sole nitrogen sources. Ethylamine is not assimilated. Growth is observed at 25 °C, but not at 30 °C. Growth on 50% (w/w) glucose-yeast extract agar is negative. Starch-like substances are not produced. Urease activity and diazonium blue B reaction are positive.

##### GenBank accession numbers.

Holotype GDMCC 2.577 (D1/D2: PX765644, ITS: PX810227).

##### Note.

Physiologically, *P. guizhouensis* can be distinguished from its close relative *P. allantoinivorans* by its assimilation of inulin, its failure to assimilate melibiose and citrate, and its lack of growth at 30 °C (Table 3).

## Discussion

In the present study, three yeast strains were isolated from plant materials collected from different regions of China during yeast diversity surveys conducted in 2022 and 2025. Based on a combination of phylogenetic analyses and phenotypic characterization, these strains are described as two novel species, namely *T. arisaematis* and *P. guizhouensis*. In addition to these two novel taxa, our sampling also recovered eight other species commonly associated with plant materials. These findings expand our knowledge of the diversity, distribution, and ecology of yeasts associated with plant materials.

Members of *Tremella* are dimorphic fungi that possess both a dikaryotic filamentous stage and a haploid unicellular yeast stage (Bandoni and Boekhout 2011; Zhao et al. 2019). Ecologically, the filamentous morphs are mainly parasitic on or associated with other fungi or lichens (Chen 1998; Bandoni and Boekhout 2011; Zhao et al. 2019; Fan et al. 2021). By contrast, the yeast morphs exhibit a broader substrate range, having been recovered from leaves, phytotelmata, fruits, dead wood, tree trunks, plant residues, and animal sources; however, they are most frequently isolated from basidiocarps on decayed wood (Bandoni and Boekhout 2011; Malysheva et al. 2015; Khunnamwong et al. 2019; Zhao et al. 2019; Navarro et al. 2022). Interestingly, *T. yokohamensis* was isolated not only from a plant substrate, namely the trunk of *Eucalyptus* sp., but also from an animal source, the nostril of a koala bear (Alshahni et al. 2011). In the present study, the novel species *T. arisaematis* was isolated from leaves, beech litter, and soil collected from diverse geographic regions, including China, Austria, and Japan. The isolates from Japan, however, might represent incidental occurrences, as most *Tremella* species are typically associated with plant material. Neither basidiospores nor basidiocarps were observed in the two strains of *T. arisaematis* examined here. This may be due to incompatibility between the available strains or to unsuitable culture conditions and substrates. However, the sexual state, including basidiocarp formation, may be discovered when compatible strains are obtained and paired under appropriate conditions and on suitable substrates, as reported for *T. yokohamensis* (Malysheva et al. 2015; Kaeoniwong et al. 2024). To gain a deeper understanding of the growth and life cycle of this novel species, we propose future experiments in which dikaryotic hyphae obtained from mating compatible strains will be maintained on a suitable nutrient medium and subsequently induced to form fruiting bodies.

Species of *Pseudotremella* are widely distributed and have been recovered from a variety of habitats. Their sexual morphs have primarily been observed on natural substrates (Bandoni and Boekhout 2011), whereas the yeast morphs have been isolated from decaying fungi, tree bark, lichens, leaves, and animal sources (Middelhoven 2005; Satoh et al. 2013; Jiang et al. 2024; Pérez et al. 2024; Feng et al. 2025), with most records showing an association with plant materials. In the present study, we isolated a yeast strain of *P. guizhouensis* from rotting wood. Moreover, three additional strains recovered from various substrates in geographically distant regions were also identified as belonging to this novel species, suggesting that *P. guizhouensis* is relatively common and widely distributed across diverse localities.

## Supplementary Material

XML Treatment for Tremella arisaematis

XML Treatment for Pseudotremella guizhouensis

## Additional information

### Artificial Intelligence (AI) use

The authors accept full responsibility for the content of the manuscript, including the disclosure of any use of AI.

No AI tools were used in the preparation of this manuscript.

### Author ORCIDs

L. Tian https://orcid.org/0009-0000-5260-9107

C.-Y. Chai https://orcid.org/0000-0001-7753-6223

Q.-H. Niu https://orcid.org/0000-0003-1695-7117

F.-L. Hui https://orcid.org/0000-0001-7928-3055

### Data availability

All of the data that support the findings of this study are available in the main text.
